# Supplementary material for: The autophagy protein, ATG14 safeguards against unscheduled pyroptosis activation to enable embryo transport during early pregnancy
Source: bioRxiv. 2024 Oct 26:2024.03.19.585812. Originally published 2024 Mar 20. Preprint. [Version 2] doi: 10.1101/2024.03.19.585812 (PMC10983954; doi:10.1101/2024.03.19.585812)
Supplement: Supplement 1 [file media-1.pdf]

The autophagy protein, ATG14 safeguards against unscheduled pyroptosis activation to enable embryo transport during early pregnancy

Pooja Popli[1], Arin K. Oestreich[2,3], Vineet K. Maurya[4], Marina N. Rowen[2,3], Yong Zhang[5], Michael J. Holtzman[5], Ramya Masand[1], John Lydon[4], Shizuo Akira[6,7], Kelle H. Moley[2,3], and Ramakrishna Kommagani[1,8*]

***Correspondence to:**

Ramakrishna Kommagani

Associate Professor

Dept. of Pathology and Immunology

Dept. of Molecular Virology and Microbiology

Alkek Center for Metagenomics and Microbiome Research

T228A, One Baylor Plaza

Baylor College of Medicine

Houston, TX, 77030

Email: Rama.Kommagani@bcm.edu

Phone: (713)-798-5085

Fax: 713-798-5838

This PDF file includes:

1. Supplementary Figure 1: *Atg14* cKO mice show normal gross morphology of uterus and ovary.

2. Supplementary Figure 2: *Atg14* cKO mice altered autophagy markers expression.

3. Supplementary Figure 3: *Atg14* cKO mice show substantial loss of ciliary epithelial cells.

4. **Supplementary Figure 4**: Average number of embryos are unaltered in *Atg14* cKO or Polyphyllin-treated D4 pregnant mice

3. Supplementary Table 1: List of primers and TaqMan probes

4. Supplementary Table 2: List of antibodies

Supplementary Figure Legends:

Figure S1: *Atg14* cKO mice show normal gross morphology of uterus and ovary. Histological analysis using H&E staining of 8-week-old virgin *Atg14* control and cKO mice uteri and ovary. LE: luminal epithelium, GE: glandular epithelium, ST: stroma, AF: antral follicle, CL: corpus luteum.

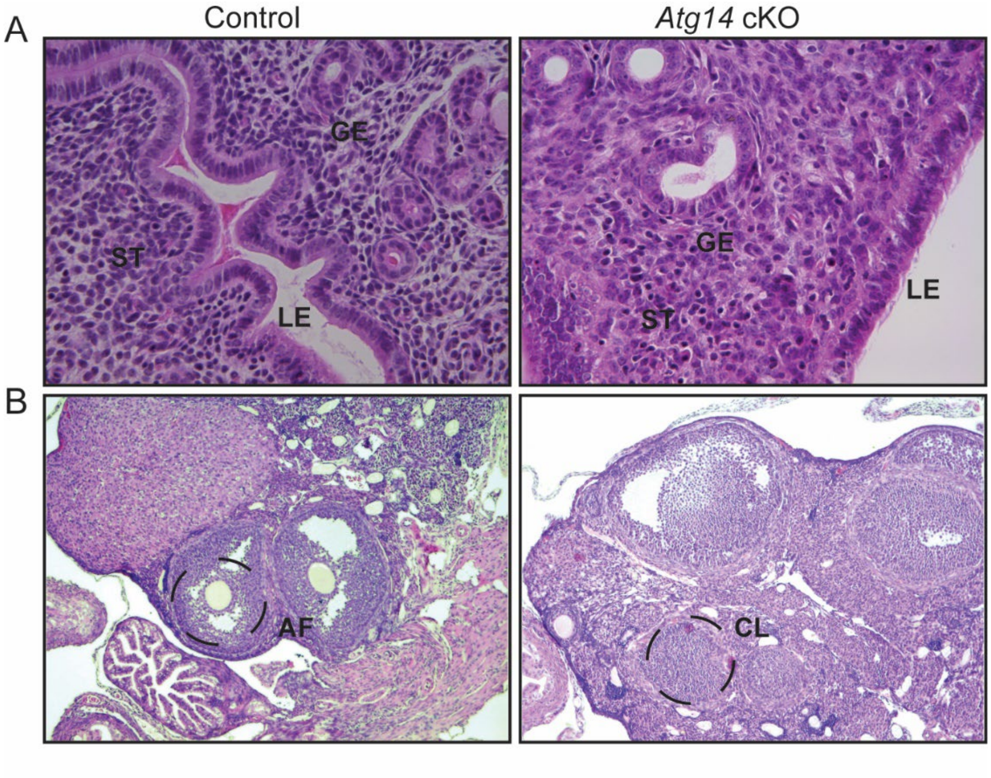

Figure S2: ***Atg14*** **cKO mice altered autophagy markers expression.** (**A**) Immunofluorescence analysis to show ATG14 expression in ampulla and isthmus regions of oviducts. LE: Luminal epithelium, M: smooth muscle. Scale bar: 100 μm; 20 μm. The upper panel shows the 10X objective images and the lower panel shows the 20X objective images. (**B**) Western blotting shows LC3B and p62 expression in control and cKO oviducts tissues. β-actin was used as a loading control.

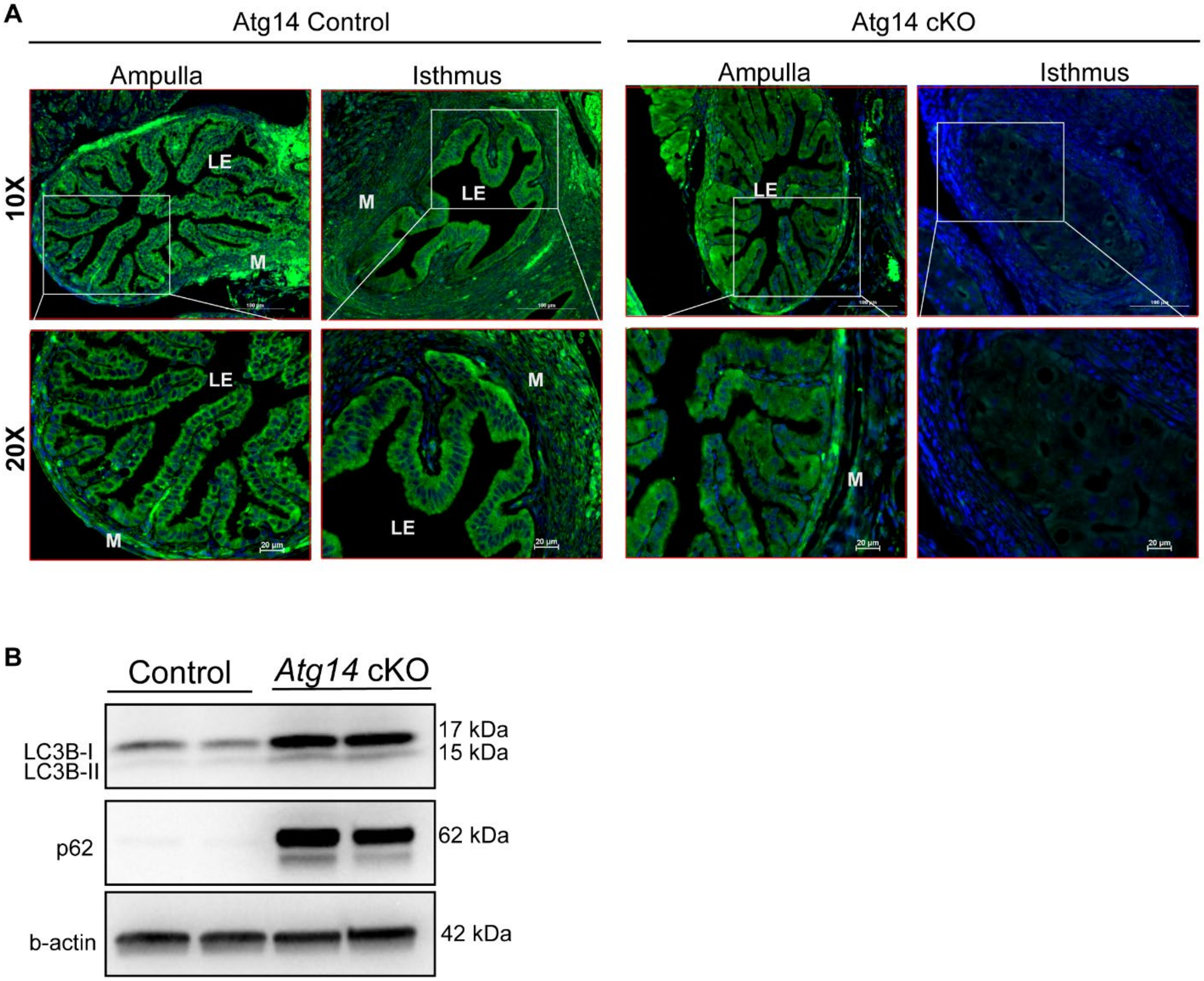

Fig. S2

Figure S3: ***Atg14* cKO mice show substantial loss of ciliary epithelial cells**. Immunohistochemistry to show FoxJ1-positive staining in *Atg14* control and cKO oviducts**.** The upper panel shows the ampullary section and the lower panel shows the isthmus section. Images were taken at 20X. Scale bar: 10μm.

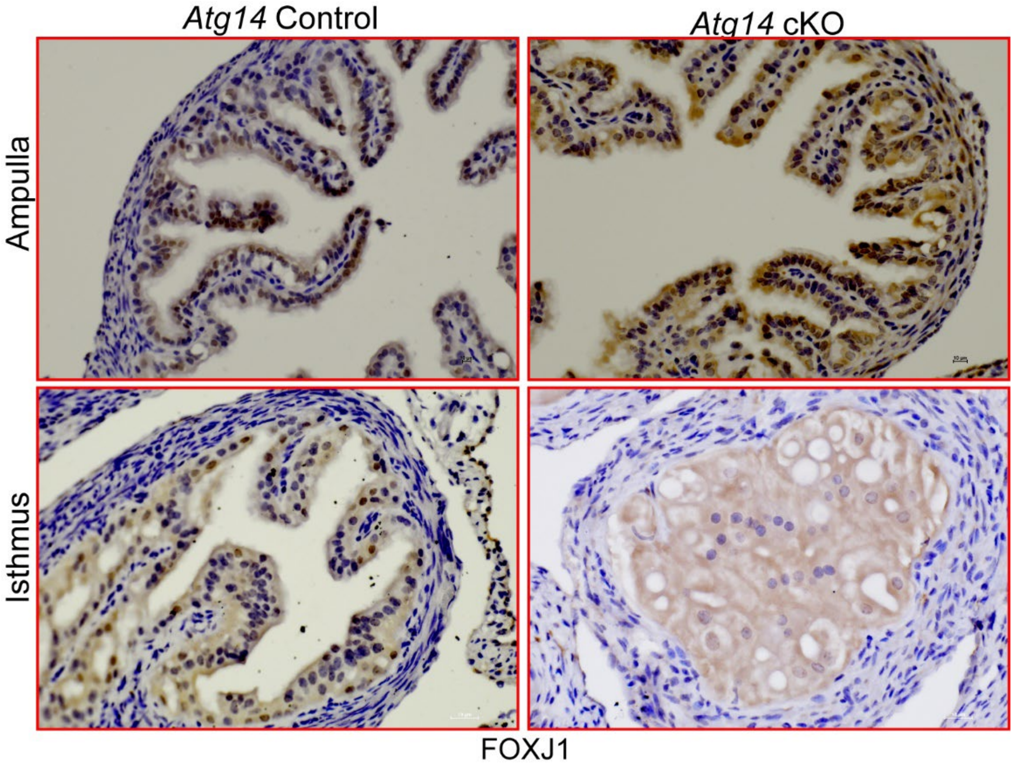

Fig. S3

Figure S4: Average number of embryos are unaltered in *Atg14* cKO or Polyphyllin-treated D4 pregnant mice. (A) Average number of embryos retrieved from *Atg14* cKO (n=5) oviducts or control uteri (n=5). **(B)** Average number of embryos retrieved from vehicle (n=3) uteri and polyphyllin-treated (n=3) D4 pregnant mice oviducts & uteri.

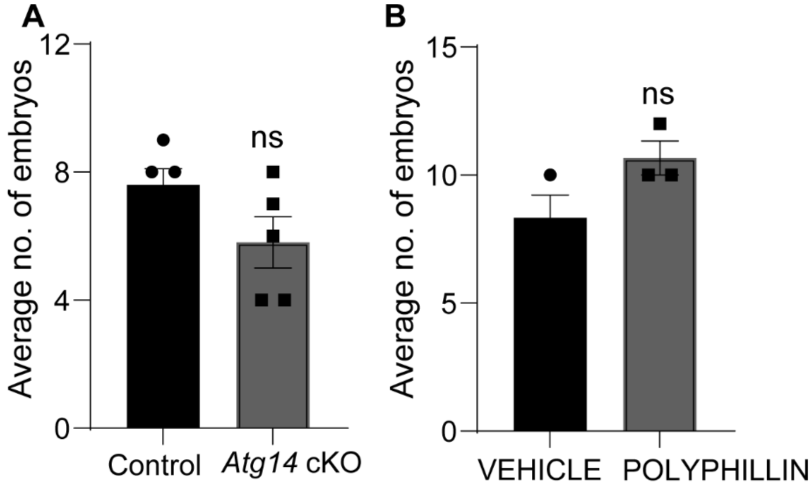

Fig. S4

Supplementary Table 1. List of primers and TaqMan probes

Gene name	Species	Application, Chemistry	Company	Sequence/Cat. No.
Atg14 f/f	Mouse	Genotyping	IDT	P1: TTGACCGTCACAGGGTGTGAGTGACTT<br>P2: AAGCAGAGTTAGGCTTCCCTGGTAGAA<br>P3: CCCATCTCCATTCCTGGATTACTGGAC<br>P4: CTAAAGCGCATGCTCCAGACTGCCTTG
PR^{Cre}	Mouse	Genotyping	IDT	P1: ATGTTTAGCTGGCCCAAA TG<br>P2: TAT ACC GAT CTC CCT GGA CG<br>P3: CCC AAA GAG ACA CCA GGA AG
$Foxj1^{Cre}$	Mouse	Genotyping	IDT	P1: ATTTGGGCCAGCTAAACATGC<br>P2: GCAAAACAGGTAGTTATTCGG
Esr1	Mouse	qPCR, TaqMan	ABI	Mm00433149_m1
Pgr	Mouse	qPCR, TaqMan	ABI	Mm00435628_m1
Atg14	Mouse	qPCR, TaqMan	ABI	Mm00553733_m1
Tnf-alpha	Mouse	qPCR, TaqMan	ABI	Mm00443258_m1
Cxcr3	Mouse	qPCR, TaqMan	ABI	Mm99999054_s1
Lif	Mouse	qPCR, TaqMan	ABI	Mm00434761_m1
Mcm2	Mouse	qPCR, TaqMan	ABI	Mm00484804_m1
Ccnd1	Mouse	qPCR, TaqMan	ABI	Mm00432359_m1
Fgf18	Mouse	qPCR, TaqMan	ABI	Mm00433286_m1
ATG14	Human	qPCR, TaqMan	ABI	Hs00208732_m1
18S	Human<br>Mouse	qPCR, TaqMan	ABI	4318839

*All primer sequences are written 5’ to 3’

ABI-applied biosystems

IDT-integrated DNA technologies.

Supplementary Table 2. List of antibodies

Antibody	Company, Catalogue number	Application
ATG14	Proteintech, 24412-1-AP	Immunofluorescence
GSDMD	Thermo Fisher Scientific, Cat# PA5-115330	Immunoblotting
GSDMD	Abcam, ab209845	Immunofluorescence
CASPASE 1	Abcam, ab138483	Immunoblotting
Ki-67	Abcam, ab15580	Immunofluorescence
FOXJ1	Sigma, HPA 005714	Immunohistochemistry
PAX8	CST, #59019s	Immunohistochemistry
MUC1	Abcam, ab15481	Immunofluorescence
TOM20	ab186735	Immunofluorescence
CYTOCHROME C (6H2.B4)	Thermo Fisher Scientific, 33-8200	Immunofluorescence
Alpha-smooth muscle actin (KRT8)	Developmental Studies Hybridoma Bank, TROMA-I	Immunofluorescence
Normal Rabbit IgG	CST, #2729	Immunofluorescence
Goat anti-Rat IgG (H+L) Cross-Adsorbed Secondary Antibody, Alexa Fluor™ 488	Thermofisher Scientific, A11006	Immunofluorescence
Goat anti-Rabbit IgG (H+L) Highly Cross-Adsorbed Secondary Antibody, Alexa Fluor™ 488	Thermofisher Scientific, A11034	Immunofluorescence
Goat anti-Rabbit IgG (H+L) Highly Cross-Adsorbed Secondary Antibody, Alexa Fluor™ 594	Thermofisher Scientific, A11037	Immunofluorescence
GAPDH	CST, #2118S	Immunoblotting
Anti-rabbit IgG, HRP-linked	CST, #7074	Immunoblotting
